# Interaction of lecithin:cholesterol acyltransferase with lipid surfaces and apolipoprotein A-I-derived peptides[S]

**DOI:** 10.1194/jlr.M082685

**Published:** 2018-02-08

**Authors:** Marco G. Casteleijn, Petteri Parkkila, Tapani Viitala, Artturi Koivuniemi

**Affiliations:** Division of Pharmaceutical Biosciences, Faculty of Pharmacy, University of Helsinki, Helsinki, Finland

**Keywords:** high density lipoprotein, lipoproteins, lipid membranes

## Abstract

LCAT is an enzyme responsible for the formation of cholesteryl esters from unesterified cholesterol (UC) and phospholipid (PL) molecules in HDL particles. However, it is poorly understood how LCAT interacts with lipoproteins and how apoA-I activates it. Here we have studied the interactions between LCAT and lipids through molecular simulations. In addition, we studied the binding of LCAT to apoA-I-derived peptides, and their effect on LCAT lipid association-utilizing experiments. Results show that LCAT anchors itself to lipoprotein surfaces by utilizing nonpolar amino acids located in the membrane-binding domain and the active site tunnel opening. Meanwhile, the membrane-anchoring hydrophobic amino acids attract cholesterol molecules next to them. The results also highlight the role of the lid-loop in the lipid binding and conformation of LCAT with respect to the lipid surface. The apoA-I-derived peptides from the LCAT-activating region bind to LCAT and promote its lipid surface interactions, although some of these peptides do not bind lipids individually. The transfer free-energy of PL from the lipid bilayer into the active site is consistent with the activation energy of LCAT. Furthermore, the entry of UC molecules into the active site becomes highly favorable by the acylation of SER181.

Coronary heart disease (CHD) and its comorbidities are global health threats showing an increased prevalence in industrial as well as in developing countries. Although many drugs for treating CHD exist, such as the cholesterol (CHOL)-lowering statins, a substantial residual vascular risk remains (1). According to the Residual Risk Reduction Initiative, this represents a paramount public health challenge in the twenty-first century (1). Thus, more effective medical strategies are needed to slow down and reverse this trend. Low HDL cholesterol (HDL-C) and elevated triglyceride levels are attributed as the two key contributors to the high residual CHD risk (2). Therefore, in the last decade, an increased focus has been on elevating the plasma concentration of HDL-C as a generally accepted intervention to prevent or reduce the development of CHD (3, 4), as opposed to the lowering of high LDL cholesterol (LDL-C), which in turn accelerates the fat accumulation into arterial walls. Traditionally, a high HDL-C concentration in blood has been regarded as a preventive measure reflecting the ability of HDL particles to transport CHOL from the peripheral tissues back to the liver, including the mainly LDL-derived CHOL from the arterial walls. This process is termed reverse CHOL transport (RCT) (5, 6). However, there is a growing body of literature showing that the elevation of the HDL-C by niacin or cholesterol ester (CE) transfer protein inhibitors does not, or only moderately improves cardiovascular events (7–9). In addition, recent genetic association studies have raised doubts toward the HDL-C hypothesis and causality of HDL-C in the development of CHD (9–11). The failure of a large number of HDL-C-raising therapies and the findings of genetic studies have led to a significant uncertainty concerning the benefit of raising HDL-C levels in the treatment of CHD. Still, several epidemiological studies support an inverse correlation between HDL-C and CHD (9, 12, 13). Hence, the in-depth understanding of the structure-function relationship of HDL particles and the enzymes processing them at the molecular level is all the more important for treating CHD.

LCAT (EC 2.3.1.43) is one of the key enzymes driving the maturation of nascent HDL particles in serum (14). Specifically, LCAT is responsible for linking the acyl chain cleaved from a phospholipid (PL) to an unesterified cholesterol (UC) molecule resulting in a CE molecule, which in turn transforms discoidal HDL particles to spherical ones, which is a crucial step in RCT (15, 16). Essentially, the esterification of UC molecules increases the CHOL loading capacity of HDL particles, enabling a more efficient RCT. In addition to its importance in RCT, mutations in the *LCAT* gene result in metabolic disorders, such as familial LCAT deficiency and fish-eye disease in which the body’s ability to metabolize CHOL is severed, leading to corneal lipid deposition, hemolytic anemia, and finally renal failure (17–19). However, despite the fact that LCAT deficiencies are associated with low HDL-C and esterified CHOL levels, the role LCAT deficiency in the development of atherosclerosis is unclear (20).

The initial step in the reaction cycle of LCAT is its binding to a lipoprotein surface. Previous research studies have established that apolipoproteins, especially the principal LCAT activator, apoA-I, play a negligible role in the attachment rate of LCAT to lipoprotein surfaces, but decrease its detachment rate from surfaces (21, 22). This suggests that LCAT initially binds to the lipid moiety of lipoprotein particles, which is then followed by interactions with apoA-I promoting stronger binding and activation. However, the specific amino acids responsible for initially anchoring LCAT to lipoprotein surfaces have not been revealed, although deletion mutant studies have shown that amino acids 53-71 and the disulphide bond formed between cysteines 50 and 74 of LCAT are crucial for the interfacial recognition of LCAT (22–24). Furthermore, it was shown by Murray et al. (25) that when LCAT was bound to HDL or hydrophobic surfaces during sink immunoassays, the epitopes of the 121-136 region were not accessible for antibodies. Moreover, it is not known how deep the LCAT is buried into the lipid matrix, whether a membrane binding region has specific interactions with different lipids that could facilitate its activity, and how LCAT is oriented with respect to lipoprotein surfaces when bound and not bound to apoA-I. The next steps in the reaction cycle are the apoA-I driven activation of LCAT by an unknown mechanism and the diffusion of PLs and UC to the active site (14, 26). Consequently, the sn-2 chain of PLs is preferentially lipolyzed and the acyl-intermediate of LCAT is formed in which the acyl chain is covalently bound to SER181, which is a part of the ASP-HIS-SER catalytic triad also found in other lipases belonging to the α/β hydrolase family (14, 27–29). Next, the acyl chain bound to SER181 is transferred to UC. Finally, the newly synthesized CE molecule diffuses from the active site into the lipoprotein. While the LCAT activator region of apoA-I (central helixes 5, 6, and 7, or residues 121-142, 143-164, and 165-186, respectively) is roughly known based on the apoA-I LCAT deficiency mutations and a vast amount of experimental data, the more specific LCAT interaction site and role of this in the different reaction steps remains unclear (26, 30, 31). By revealing the mechanisms behind the different reaction steps of LCAT, the way may be paved for inventing novel positive allosteric modulators of LCAT that aim to raise HDL-C in a manner that would be beneficial for the treatment of CHD.

For years, investigations concerning the LCAT lipid bilayer interaction and activation by apoA-I have been hindered by a lack of the detailed atomistic structure of LCAT. Recently, however, X-ray structures of LCAT have become available enabling, for example, the computational studies of LCAT interacting with lipid surfaces and apoA-I to elucidate the mechanistic details concerning the CHOL esterification process (32–35). From the structures it is evident that the tertiary and secondary structure of LCAT is similar to lysosomal phospholipase A2, as pointed out by three similar folds: the cap domain, the membrane-binding domain, and the α/β hydrolase domain (see Fig. 1A) (35). Most importantly, structural details reveal that LCAT possesses a lid-loop that can move aside from the tunnel opening enabling the entry of lipids into the active site where the catalytic triad is located, thus being consistent with other lipases (32, 34).

In this study, we carried out several atomistic and coarse-grained (CG) molecular dynamics simulations to investigate the effects of different conformational states of LCAT to its interaction with a lipid bilayer comprised of dioleoylphosphatidylcholines (DOPCs) and UC molecules. To gain insights into apoA-I-mediated LCAT activation, we utilized quartz-crystal-microbalance (QCM) and multi-parametric surface plasmon resonance (MP-SPR) experiments to investigate the effect of apoA-I-derived peptides on the binding of LCAT to lipid bilayers, and the binding of LCAT to these peptides, respectively. These experiments were complemented with extensive free-energy simulations to reveal the role of the secondary structure of apoA-I-derived peptides in lipid interactions. Finally, we studied the energetics of lipid ligand entry to the active site of LCAT.

Our simulations highlight the importance of specific nonpolar amino acids in LCAT-lipid interactions. In addition, we show that the membrane-anchoring nonpolar amino acids attract UC molecules adjacent to them. The results also demonstrate that the lid-loop plays an important role in the conformation of LCAT with respect to the lipid surface. Furthermore, the experiments indicate that peptides derived from the LCAT-activating region of apoA-I bind differently to LCAT and promote its lipid surface binding, although some of the peptides do not bind to lipids individually. We provide an explanation for this mechanism utilizing computational free-energy calculations. It was also found that the transfer free-energy of PL from the lipid bilayer to the active site is consistent with the activation energy of LCAT. Finally, our results indicate that the acyl-intermediate of LCAT highly facilitates the accessibility of UC molecules into the active site.

## MATERIALS AND METHODS

### Computational procedures

#### Construction of simulation systems.

The high-resolution X-ray structures of LCAT were acquired from the Brookhaven databank with the accession codes of 4XWG and 5BV7 (32, 34). In addition, one mutated residue (C31Y in 4XWG) was changed back to one matching with the native LCAT utilizing the PYMOL software (36). The 4XWG structure is considered to be the closed conformation and the 5BV7 structure the open conformation of LCAT based on the lid-loop orientation with respect to the active site tunnel opening. The MODELLER program was utilized to construct the missing lid-loop regions in both structures (37). The model building script (model-single.py) of the MODELLER program was utilized to construct the missing lid-loop regions and 10 different lid-loop structures were generated for the open and closed states. The structures with the lowest DOPE score were chosen in both cases for further modeling studies. It is noteworthy that the residues 1-20 (N terminal) and 398-422 (C terminal) are totally or partially missing from the X-ray structures. These amino acids were not added to the structure because their configuration and structure, with respect to the rest of the LCAT structure, is highly unclear and requires further experimental information. However, a very recent study indicates that the N- and C-terminal ends of LCAT play an important role in LCAT HDL surface interaction. This matter is discussed in the Discussion section in more detail.

Two LCAT-in-water systems were constructed: one for the open and one for the closed lid-loop conformation. In the rest of the article, we refer to these systems by abbreviations LCAT-water-open and LCAT-water-closed. The dimensions of LCAT-water-open and LCAT-water-closed simulation boxes were 8.5 × 7 × 7 nm. The open and closed LCAT enzymes were solvated with 12,000 water molecules. In addition, nine sodium ions were added to neutralize the simulation systems. In the construction of LCAT-lipid bilayer systems, we used a preequilibrated DOPC/UC bilayer available in the website of the Slipid developers. To construct atomistic lipid membrane systems, the LCAT was placed on the surface of the DOPC/UC bilayer so that the nonpolar residues of the tunnel-opening and the membrane binding domain were buried in the lipid matrix and the active site tunnel opening was pointing toward the lipid bilayer. This was done for both the lid-open and lid-closed LCAT structures. The DOPC and UC molecules overlapping with LCAT were removed from the systems. Following this, the systems were solvated approximately with 20,000 water molecules and nine sodium ions were added to neutralize the systems. One additional atomistic system was constructed for the open LCAT lid-loop conformation where SER181 was acylated with an oleic acid. We refer to these all-atom (AA) lipid membrane models as LCAT-mem-open-AA, LCAT-mem-closed-AA, and LCAT-mem-acyl-AA. The starting structures for the corresponding CG LCAT-lipid systems representing these three atom-scale models were constructed similarly (LCAT-mem-open-CG, LCAT-mem-closed-CG, and LCAT-mem-acyl-CG). In **Table 1**, a more detailed list of molecules in each simulation system is presented.

**TABLE 1.** Simulated systems with their molecular compositions and simulation times

System	Details	DOPC/POPC	CHOL	Water (mol/beads)	Total Simulation Time (μs)
LCAT-water-open-AA	Lid open	0	0	12,000	0.4
LCAT-water-closed-AA	Lid closed	0	0	12,000	0.4
LCAT-mem-open-AA	Lid open	286	132	20,000	1
LCAT-mem-closed-AA	Lid closed	286	132	20,000	1
LCAT-mem-acyl-AA	SER181 acylated	286	132	20,000	1
LCAT-mem-open-CG	Lid open	226	105	10,000	20*^a^*
LCAT-mem-closed-CG	Lid closed	226	105	10,000	20*^a^*
LCAT-mem-acyl-CG	SER181 acylated	226	105	10,000	20*^a^*
LCAT-mem-open-AA-DOPC-PMF	Lid open	286	132	20,000	1.6
LCAT-mem-open-AA-UC-PMF	Lid open	286	132	20,000	2.0
LCAT-mem-acyl-AA-UC-PMF	SER181 acylated	286	132	20,000	1.7
LCAT-mem-open-CG-LCAT-PMF	Lid open	226	105	10,000	96*^a^*
LCAT-mem-closed-CG-LCAT-PMF	Lid closed	226	105	10,000	96*^a^*
Pep_122-142-CG-PMF	Helix or coil	128	0	1,800	8*^a^*
Pep_135-155-CG-PMF	Helix or coil	128	0	1,800	8*^a^*
Pep_150-170-CG-PMF	Helix or coil	128	0	1,800	8*^a^*
Pep_185-205-CG-PMF	Helix or coil	128	0	1,800	8*^a^*
Pep_150-170-CG-PMF-Y166F	Helix or coil	128	0	1,800	8*^a^*

a The total simulation time of the CG systems was multiplied by four because the diffusion dynamics of MARTINI water beads is approximately four times faster compared with real water (43, 45).

#### Simulation force fields and parameters.

Molecular dynamics simulations were carried out with the GROMACS simulations package (version 5.1.2) (38, 39). The SLipids (http://www.fos.su.se/~sasha/SLipids/) and AMBER99SB-ILDN force fields were utilized for lipids and protein molecules in atomistic simulations, respectively (40–42). The MARTINI force field was used for the CG representations (43, 44). The polarizable water and protein models of the MARTINI force field were utilized (45, 46).

Concerning the atomistic simulations, all systems were first energy minimized with the steepest descent algorithm. After this, short 10 ns equilibrium simulations were carried out to stabilize pressure fluctuations that were followed by 400 ns (LCAT-water systems) or 1 μs production simulations (LCAT-mem systems). The time step was set to 2 fs, and the temperature and pressure were maintained at 310 K and 1 bar, respectively. The Berendsen coupling schemes were utilized for achieving constant temperature and pressure during the short equilibrium period of atomistic systems (47). After this, the Nose-Hoover and the Parrinello-Rahman coupling algorithms were employed for treating temperature and pressure in the atomistic systems, respectively (48, 49). The isotropic or semi-isotropic pressure coupling scheme was used in the LCAT-water and LCAT-mem systems, respectively. Water plus ions, lipids, and protein were separately coupled to heat paths. For the Lennard-Jones interactions, a cut-off of 1 nm was used, and the electrostatic interactions were handled with the particle-mesh Ewald method with a real space cut-off of 1.0 (50). The LINCS algorithm was applied to constrain covalent hydrogen bond lengths (51).

Regarding the CG simulations, the temperature and pressure were handled with the v-rescale and the Parrinello-Rahman schemes, respectively (49, 52). Coupling constants of 1 and 12 ps^−1^ were used for the temperature and pressure schemes, respectively. Lipids, protein, and water molecules were separately coupled to a heat path. The reaction-field electrostatics and Lennard-Jones interactions were employed with cut-offs of 1.1 nm. The relative electrostatic screening was set to 2.5 because the polarizable water and protein models of the MARTINI force field were used. A time step of 20 fs was used and all CG simulations were simulated up to 20 us (scaled MARTINI time). The ElNeDyn scheme was employed for LCAT (53).

#### Free-energy calculations.

For calculating the free-energy profiles for phosphatidylcholine (PC) or UC molecules entering the active site of LCAT, either a UC or a PC molecule was pulled from the lipid bilayer to the active site of LCAT in the lid-open state to generate umbrella windows (these systems are coined as LCAT-mem-open-DOPC-PMF-AA and LCAT-mem-open-CHOL-PMF-AA). The free-energy profiles for PC and UC molecules were calculated utilizing atomistic simulations. In addition, the free-energy profile for a UC molecule was calculated in the LCAT-mem systems where SER181 was acylated (LCAT-mem-acyl-UC -PMF-AA). The oxygen atom of UC or the phosphorous atom of DOPC was pulled toward the center of mass of the SER181 residue with a pull rate of 0.001 nm/ps and a force constant of 10,000 kJ/mol-nm^2^. The resulting reaction coordinate was divided into 39 umbrella windows. The force-constant of the spring was set to 2,000 kJ/mol·nm^2^. Each umbrella window was sampled up to 40–50 ns, thus the total simulation time ranged from 1.6 to 2.0 μs per system. The last 34–28 ns of the simulation trajectories were used to construct the PMF profiles.

For determining the lipid-binding free-energies for different apoA-I-derived peptides at the CG level, each peptide was pulled from the water phase to the center of the POPC bilayer with a pull rate of 0.001 nm/ps and a force-constant of 20,000 kJ/mol·nm^2^. This was followed by a generation of 20 umbrella windows along the bilayer normal. Each window was sampled up to 100 ns (400 ns in scaled MARTINI time) and the centers of mass of peptides were constrained to the center of each umbrella window by using a force constant of 500 kJ/mol·nm^2^. The last 320 ns of the simulation trajectories were used to construct the PMF profiles. To estimate the effect of secondary structure to the binding of free-energies, potential mean force (PMF) profiles were calculated for peptides fully adopting α-helical or coil secondary structures.

In addition to the above systems, the free-energy profiles for LCAT along the lipid membrane normal were also estimated. First, LCAT was pulled from the surface of DOPC-CHOL membrane to the water phase by using a constant pulling speed of 0.0002 nm/ps and a force constant of 20,000 kJ/mol·nm^2^. The pulling was done for end structures of LCAT-mem-open-CG and LCAT-mem-closed-CG simulations. Next, the reaction coordinate was divided into 30 different umbrella windows. Each window was sampled up to 0.8 μs (3.2 μs in scaled MARTINI time) in with a force constant of 1,000 kJ/mol·nm^2^. Thus, the total simulation time for each LCAT system was 96 μs (scaled MARTINI time). The last 2 μs of the simulation trajectories were used in the construction of the PMF profiles.

#### Analysis methods.

All the analysis programs reported here are part of the GROMACS simulations package unless mentioned otherwise. The gmx rmsf and gmx rmsd programs were used to produce root-mean-square fluctuation (RMSF) and root-mean-square deviation (RMSD) graphs. Only the backbone atoms of LCAT were included in the RMSD and RMSF analysis. The RMSF profiles were calculated as a function of amino acid residues of LCAT after the RMSD profiles were stabilized (after 100 ns). The secondary structure of LCAT was monitored as a function of time with the secondary structure plugin of the VMD (54). The average number of hydrogen bonds was calculated utilizing the gmx hbond analysis tool. The maximum distance between a donor and an acceptor was set to 0.35 nm, and the maximum angle formed by hydrogen bonding atoms hydrogen-donor-acceptor was set to 30 degrees. The number of salt bridges in the structure of LCAT concerning the lid-region was calculated by the gmx mindist program with a distance criterion of 0.6 nm between the charged side chain central carbon atoms of negatively and positively charged amino acids (GLU-ARG, GLU-LYS, ASP-ARG, and ASP-LYS). The solvent-accessible surface areas (SASAs) for the selected amino acids were calculated using the gmx sasa program. The distance and tilt analysis were carried out with the gmx mindist and gmx gangle programs. These analyses were conducted after the number of contacts between protein and head group atoms of DOPC were in equilibrium. For producing 2D-spatial density maps, the gmx densmap program was used. The XY-planes of simulation systems were divided into 100 × 100 or 36 × 36 square bins, in which the number of UC atoms was calculated in each time frame and summed over the whole simulation time to produce number density profiles. Before the calculation, the membrane puncturing region of LCAT (the center of mass of residue PHE67) was placed to the center of the simulation box. The gmx_wham program was utilized to derive the PMF profiles from the umbrella sampling simulations. The bootstrapping method with 50 bins was used to estimate the errors for the PMF profiles. All visualizations in this work were made with either the Python codes or the VMD visualization package (54).

### Experimental procedures

#### Materials.

POPC dissolved in chloroform (25 mg/ml) was obtained from Avanti Polar Lipids (Alabaster, AL). Calcium chloride, HEPES, CHAPS, sodium chloride, EDTA, sodium azide, and potassium dihydrogen phosphate were obtained from Merck Sigma-Aldrich (Darmstadt, Germany). Potassium chloride was obtained from Honeywell Riedel de Haën (Seelz, Germany); disodium hydrogen phosphate was obtained from Fisher Scientific (Hampton, NH). Peptides and N-terminal biotin-labeled peptides were synthesized by Peptide Protein Research Ltd., Hampshire, UK and had the following amino acid sequences: *i*) 122-142, LRAELQEGARQKLHELQEKLS; *ii*) 135-155, HELQEKLSPLGEEMRDRARAH; *iii*) 150-170, DRARAHVDALRTHLAPYSDEL; *iv*) 150-170-Y166F, DRARAHVDALRTHLAPFSDEL; and *v*) 185-205, GGARLAEYHAKATEHLSTLSE. Lyophilized glycosylated human LCAT protein (Sino Biological Inc., Beijing, China; a recombinant protein produced in human cells) was reconstituted with 400 μl of ultrapure sterile water to yield LCAT at 5 μM in PBS (manufacturer specifications). Ultrapure water used for preparation of the buffer and in all measurements was prepared with a Milli-Q purification system, having a resistivity of 18 MΩ·cm and TOC level of <5 ppm. The following buffers were used: 20 mM PBS, 1 mM EDTA, 1 mM Na azide (pH 7.4) (buffer A); and 20 mM HEPES and 150 mM NaCl (pH 7.4) (buffer B).

#### The MP-SPR measurements.

Measurements were performed with a multi-parameter SPR Navi™ 200 (BioNavis Ltd., Tampere, Finland) instrument. The setup was equipped with two incident laser wavelengths, 670 nm and 785 nm, two independent flow channels, inlet tubing and outlet (waste) tubing, and an autosampler. Both of the flow channels were measured simultaneously with 670 nm and 785 nm incident light. The measurement temperature was kept constant at 293° K. Biotin-coated SPR sensors (Bionavis Ltd.) were placed in the flow-cell. The flow rates used for the streptavidin interaction with biotin, biotin-peptides with streptavidin, and for LCAT interaction with immobilized peptides were 10 μl/min. SPR spectra were recorded after introduction of buffer A into the flow-cell for 10–30 min until a stable baseline was achieved, after which the surface was fully saturated with streptavidin during a 10 min serial injection at 1.9 μM to saturate the SPR sensor. It was observed that after 5 min the SPR sensor was fully coated with streptavidin; therefore, the method was adapted to 5 min. In the second phase of the measurement, in DMSO solubilized biotin-peptides with a final concentration of 100 μM were injected in parallel into both flow channels for 7 min at 10 μM to cover the SPR sensor fully, i.e., until all streptavidin bindings sites were fully occupied. Parallel injections ensured that two peptides were analyzed simultaneously. Increasing concentrations of LCAT were then injected into both channels via serial injection, to ensure the lowest variation of LCAT injection for 15 min at increasing concentrations (156, 313, 625, 1250, 2,500, and 5,000 nM) to determine the binding constant of the LCAT-peptide complexes. The data was fitted in OriginPro (v. 8.6, OriginLab Corp., Northampton, MA) according to the Langmuir model to obtain the dissociation constants (*K_d_*) and the responses of saturated binding (*R_max_*):

$$R=\frac{R_{max}}{1+K_{d}/c}$$

where *R* is the final response after each injection and *c* is the concentration of LCAT in each injection.

#### The QCM measurements.

Prior to the QCM measurements, the PBS buffer formed after reconstitution of LCAT was exchanged for buffer A due to the extremely high sensitivity of the QCM method to slight differences in the ionic strength of the background buffer. The LCAT solution was placed in an Amicon Ultra-0.5 10K centrifugal filter device (Merck Millipore Ltd., Tullagreen Carrigtohill, Ireland) and equal volumes of buffer A were filtered through 10 times at 14,000 *g*, after which 400 μl of buffer A were added to fully recover LCAT at 5 μM. The peptides were dissolved into buffer A at a final concentration of 100 μM.

An impedance-based QCM instrument (KSV Instruments Ltd., Helsinki, Finland) was used for the measurements. The measurement temperature was kept constant at 293° K. Before measurements, silica-coated QCM sensors (Q-Sense Inc./BiolinScientific, Västra Frölunda, Sweden) were first flushed with 70% ethanol and ultrapure water, dried under nitrogen flow, and finally oxygen plasma treated (PDC-002; Harrick Plasma, Ithaca, NY) for 5 min at 29.6 W and 133–173 Pa. Samples were injected into the measurement chamber with a peristaltic pump system (Ismatec/Cole-Parmer GmbH, Wertheim, Germany). Small unilamellar POPC liposome vesicles, which were used to form supported lipid bilayers (SLBs) inside the QCM measurement chamber, were made using the thin-film hydration method followed by extrusion (11 times) through a 50 nm polycarbonate filter membrane at 333° K. The resulting small unilamellar vesicles (10 mg/ml in buffer B) had a mean number average particle size of 65 ± 7 nm and a polydispersity index of 0.167 ± 0.008 (determined from three individual measurements by a Zetasizer APS instrument; Malvern Instruments Ltd., Worcestershire, UK).

After the baseline of the signal at different frequency overtones (3, 5, and 7) was stabilized with buffer B, SLBs were formed by flowing liposome solution (0.15 mg/ml in buffer B + 5 mM CaCl_2_ at 250 μl/min) over the crystal surface for 5 min. Afterwards, buffer A was injected and the signal was left to stabilize. An additional injection of ultrapure water was added to ensure the complete formation of an SLB and absence of intact vesicles on the measurement surface. The overlap of the changes in normalized overtone frequencies (−26 Hz) was considered to be a confirmation of the presence of a good-quality SLB. The flow speed was reduced to 50 μl/min and the measurements were performed by injecting peptides at 100 μM in buffer A, LCAT at 37.5 nM in buffer A, or a combination of LCAT and peptide, through the measurement chamber for a duration of 10 min. Between each measurement, sensors were cleaned in situ by sequential 2 min injections of 20 mM CHAPS, 2% Hellmanex, 70% ethanol, and ultrapure water.

Data analysis was performed using Origin Pro (v. 8.6, OriginLab Corp., Northampton, MA). For each measurement, frequency overtone signals (3, 5, and 7) were normalized, averaged, and baseline corrected. Neither LCAT nor peptides induced changes in viscoelastic properties of the bilayer, which was seen as negligible changes in the recorded energy dissipation.

## RESULTS

### The flexible lid-loop covers nonpolar amino acids located at the tunnel opening of LCAT from water in the closed state

**Fig. 1.**
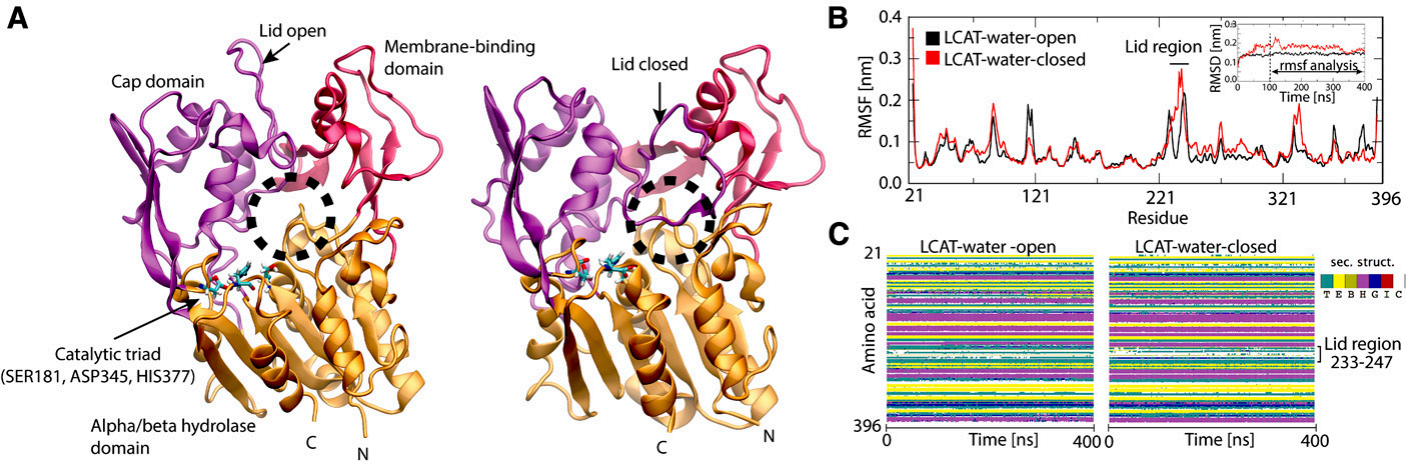
A: Structures of LCAT in the lid-open (left) and -closed (right) states rendered as cartoon models. The cap domain is colored with purple, the α/β hydrolase domain with orange, and the membrane-binding domain with red. The tunnel opening of the active site is marked with a dashed black sphere. The catalytic triad residues: SER181, ASP345, and HIS377 are rendered with sticks and colored according to element types. Cyan atoms are carbon, blue nitrogen, white hydrogen, and red oxygen atoms. B: RSMF profiles for the lid-open and -closed states. RMSD results are shown in the inset. C: Secondary structure profiles for the lid-open and -closed states.

To explore the structural and dynamic properties of the lid-loop region of LCAT, we carried out molecular dynamics simulations for LCAT in the lid-open and lid-closed states in water (**Fig. 1A**). In this fashion, we strived to establish mechanistic insights to the conformational switching of the lid-loop that, presumably, is a prerequisite for the esterification reaction of LCAT to take place. Moreover, the overall stability of the LCAT structure was assessed against the X-ray structures to validate our computational models (32–34). To construct applicable LCAT models for these purposes, the lid-loop region (amino acids 233-247) was computationally grafted to the high resolution X-ray structures of LCAT representing either the open or closed state (32, 34). During this work, another high-resolution X-ray structure of LCAT was published (Protein Data Bank accession code: 5TXF) in the lid-closed conformation with only two amino acid residues missing from the lid-loop region (33). This provided us an excellent means to validate our grafted LCAT model in the closed state. As shown in supplemental Fig. S1, our model is in good agreement with the published LCAT structure in the closed state. The produced LCAT models were simulated up to 400 ns in water surroundings. These simulation systems were coined as LCAT-water-open-AA and LCAT-water-closed-AA systems (see more details in the Materials and Methods).

First, the simulation trajectories were utilized to investigate the conformational stability, dynamics, and secondary structure changes of LCAT. As it stands out from Fig. 1A, the lid-loop exposes the active site for the entry of lipid ligands in its open state and shields the active site from water in the closed state. The RMSD profiles in Fig. 1B (inset) indicate that the backbone atoms of LCAT stabilized after 50 ns and only small deviations from the initial structures occurred. This was reflected by the average RMSDs of 0.14–0.17 nm compared with the initial backbone atom scaffolds. By examining the mobility of LCAT as a function of residue number (RMSF profiles in Fig. 1B), it was found that the lid-loop region (amino acids 233-247) showed the highest conformational fluctuations compared with the other structural parts of LCAT when neglecting the first few N- and C-terminal residues. This was an expected result. Concerning the secondary structure of LCAT (Fig. 1C), the analysis showed a stable structure without significant changes during the simulations. A closer inspection revealed that the secondary structure of the lid-loop was random-coil-rich in both conformational states, agreeing with the RMSF profiles that showed a high flexibility for this region.

**Fig. 2.**
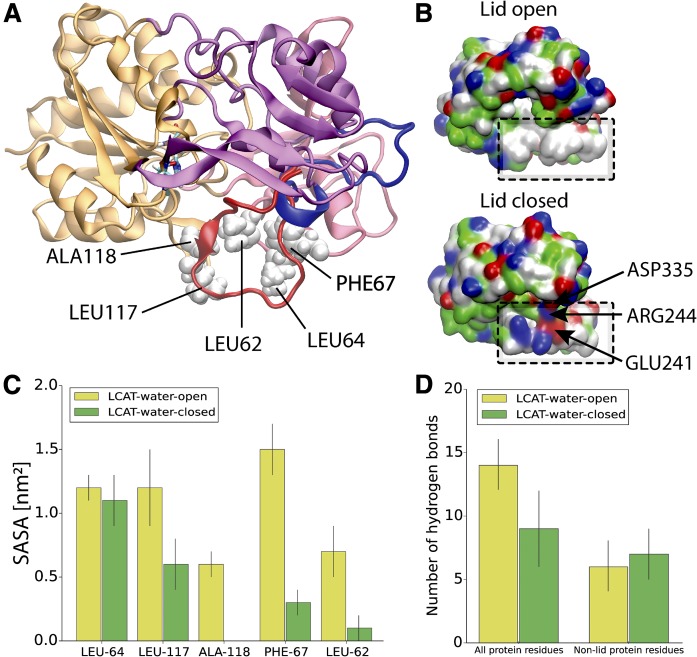
A: Structure of LCAT showing the hydrophobic amino acids located at the tunnel opening of the active site. LCAT is rendered with a cartoon representation and the domains are colored as in Fig 1. Additionally, the lid region of LCAT is colored with blue (the open state) or red (the closed state). Nonpolar amino acids interacting with the lid-loop in the closed state are labeled with the corresponding residue names and rendered with white van der Waals spheres. B: The surface presentations for LCAT in the lid-open and lid-closed conformations. The location of the hydrophobic region is marked with black and dashed squares. Red indicates negatively charged, blue positively charged, green polar, and white hydrophobic amino acid residues. The salt bridges formed by the lid-loop are marked with arrows. C: The average SASAs for the selected nonpolar amino acids in the lid-open and -closed states. D: The average number of hydrogen bonds between the lid-loop and all protein residues or non-lid protein residues in the lid-open and -closed states. Errors bars are standard deviations.

Next, we analyzed the surface properties of LCAT and SASAs for the hydrophobic amino acids located at the active site tunnel opening (**Fig. 2A**) because we hypothesized that the lid-loop shields these amino acids before LCAT detaches from the lipoprotein surface to the water phase. As shown in Fig. 2B, the surface representations of LCAT as a function of residue types (nonpolar, polar, and charged) reveal that hydrophobic amino acids located at the tunnel opening of the active site are exposed to the water phase in the open state and shielded from the water in the closed state. This result was supported by SASA calculations (averages from the last 300 ns) indicating that four of five hydrophobic residues located at the active site tunnel opening became shielded from the water in the closed state (Fig. 2C). More specifically, these amino acids were LEU62, PHE67, LEU117, and ALA118.

Considering the previous finding that hydrogen bonds and salt bridges play a role in the lid-loop conformational switching of pancreatic lipase (55), we also evaluated the average number of hydrogen bonds and salt bridges formed in the lid-closed and -open states. First, we calculated the average number of hydrogen bonds formed between the lid-loop and all LCAT amino acids. Second, we analyzed the average number of hydrogen bonds formed between the lid-loop and the non-lid-loop amino acids of LCAT. The results revealed that the lid-loop forms five internal hydrogen bonds when the conformational change from the closed to the open state takes place (Fig. 2D). However, the salt-bridge analysis indicated the breakage of two salt bridges formed by ARG244-ASP335 and ARG244-GLU241 at the same time (Fig. 2B). Our analysis did not register stable salt bridges between the lid-loop and the non-lid-loop residues in the lid-open state. In addition, no internally formed lid-loop salt bridges were found in the open state.

### The lid-open conformation enables a deeper burial of the tunnel-opening nonpolar amino acids of LCAT at lipid surfaces

In the previous section, we established that LCAT exposes the nonpolar amino acids located at the tunnel opening to water while the lid is open. Next, we asked whether these nonpolar amino acids could also interact with lipid bilayers resembling the lipid moiety of discoidal HDL particles. Additionally, we hypothesized that the lid-closed conformation also prevents the burial of the tunnel-opening nonpolar amino acids to a lipid matrix, which might be a prerequisite for the entry of lipid ligands in addition to the lid-open state. Therefore, we carried out atomistic and CG molecular dynamics simulations of LCAT to investigate how these nonpolar amino acids interact with a bilayer composed of DOPCs and CHOL. LCAT was initially placed on the surface of an equilibrated bilayer in a way that enabled the interaction of the tunnel-opening nonpolar residues with the lipid matrix. Both the lid-closed and -open states of LCAT were modeled. The atomistic simulations (termed as LCAT-mem-open-AA and LCAT-mem-closed-AA) enabled us to study the atom-scale interactions between LCAT and individual lipids in a microsecond time window. On the other hand, the CG representations (LCAT-mem-open-CG and LCAT-mem-closed-CG) extended the time window up to several microseconds, rendering it possible to estimate, e.g., the free-energy of binding of LCAT to a lipid bilayer in lid-open and -closed states utilizing umbrella sampling simulations.

**Fig. 3.**
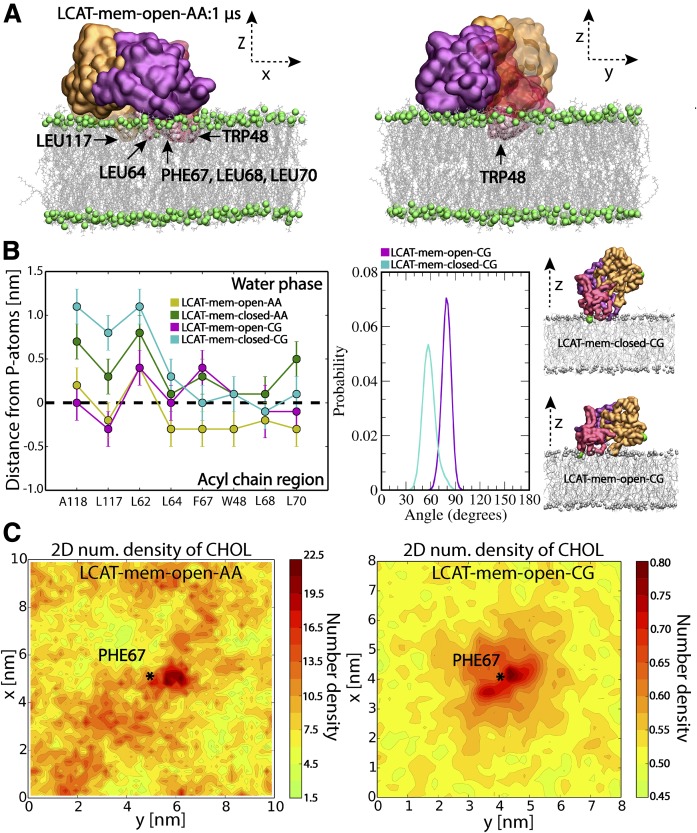
A: Snapshots from the end of LCAT-mem-open-AA simulation (1 μs). The coloring of LCAT domains is the following: orange is the α/β hydrolase domain, purple the cap domain, and red the membrane-binding domain. Gray sticks represent DOPC molecules and green spheres phosphorous atoms of DOPC. Water molecules have been removed from the snapshots for clarity. The membrane-penetrating hydrophobic residues of LCAT are marked and labeled to the snapshots. B: The average center of mass distances from the phosphorous atoms of DOPCs for the lipid-buried nonpolar amino acids in the lid-open and lid-closed membrane simulations (left); the average tilt angle of LCAT with respect to the normal of lipid membrane in LCAT-mem-open-CG and LCAT-mem-closed-CG simulations (right). In addition, snapshots approximating the average tilt angle in each case are shown. Coloring is the same as in A, but the tilt vector forming amino acids (ASN131 and MET49) has been marked with green spheres. C: The 2D-number density maps for UC in LCAT-mem-open-AA and LCAT-mem-open-CG simulations. The center of mass of PHE67 is marked by a star showing the location of the membrane-penetrating region of LCAT.

Simulation trajectories revealed that LCAT stays at the surface of the lipid bilayer up to 1 or 20 μs in atomistic and CG simulations, respectively (see supplemental Movies S1–S4). What stands out from the trajectories is that the nonpolar amino acids of the tunnel opening and the membrane-binding region stay buried in the lipid matrix in LCAT-mem-open-AA and LCAT-mem-open-CG simulations (**Fig. 3A**; supplemental Movies S1, S2). However, the lid-closed conformation prevents the deeper burial of tunnel-opening nonpolar amino acids to the lipid matrix (supplemental Movies S3, S4). Closer inspection by utilizing distance calculations with respect to the phosphorous atoms of DOPCs revealed that the hydrophobic amino acids, L117, L64, F67, W48, L68, and L70, were clearly located in the acyl chain region of PLs in LCAT-mem-open-AA simulation (see the left panel in Fig. 3B). From the distance analysis, we can see that these amino acids became less buried in the lipids in the lid-closed conformation, especially amino acids A118, L117, L64, F67, W48, L68, and L70 (see also supplemental Movie S3). As shown in Fig. 3B, this trend was also seen in CG simulations, further verifying our initial hypothesis that the lid configuration plays an important role in regulating the interaction mode of LCAT with lipids. Further analysis revealed that the lid-closed state also changes the orientation of LCAT with respect to the lipid bilayer surface, which explains the decreased burial of A118, L117, and L64 located at the hydrophobic tunnel opening. This is shown by a higher average tilt angle of LCAT calculated between the vector formed by the C_α_-atoms of MET49 and ASN131, and the lipid bilayer normal (Fig. 3B; supplemental Movies S2–S4).

Next, we asked whether the tunnel opening and membrane anchoring regions could interact with DOPC or UC molecules in a more specific way. To characterize this, we analyzed the 2D-number densities by first dividing the XY-plane into 100 × 100 or 36 × 36 squares depending on the system studied, atomistic or CG representation, respectively. This was followed by the calculation of the number densities of DOPC and UC atoms in each square (See more details in the Materials and Methods). We found no specific binding in the case of DOPC molecules, but UC molecules preferred to accumulate next to the membrane-penetrating region of LCAT in both atomistic and CG simulations (Fig. 3C; supplemental Fig. S2).

### LCAT interaction region-derived peptides of apoA-I facilitate the binding of LCAT to a lipid surface

**Fig. 4.**
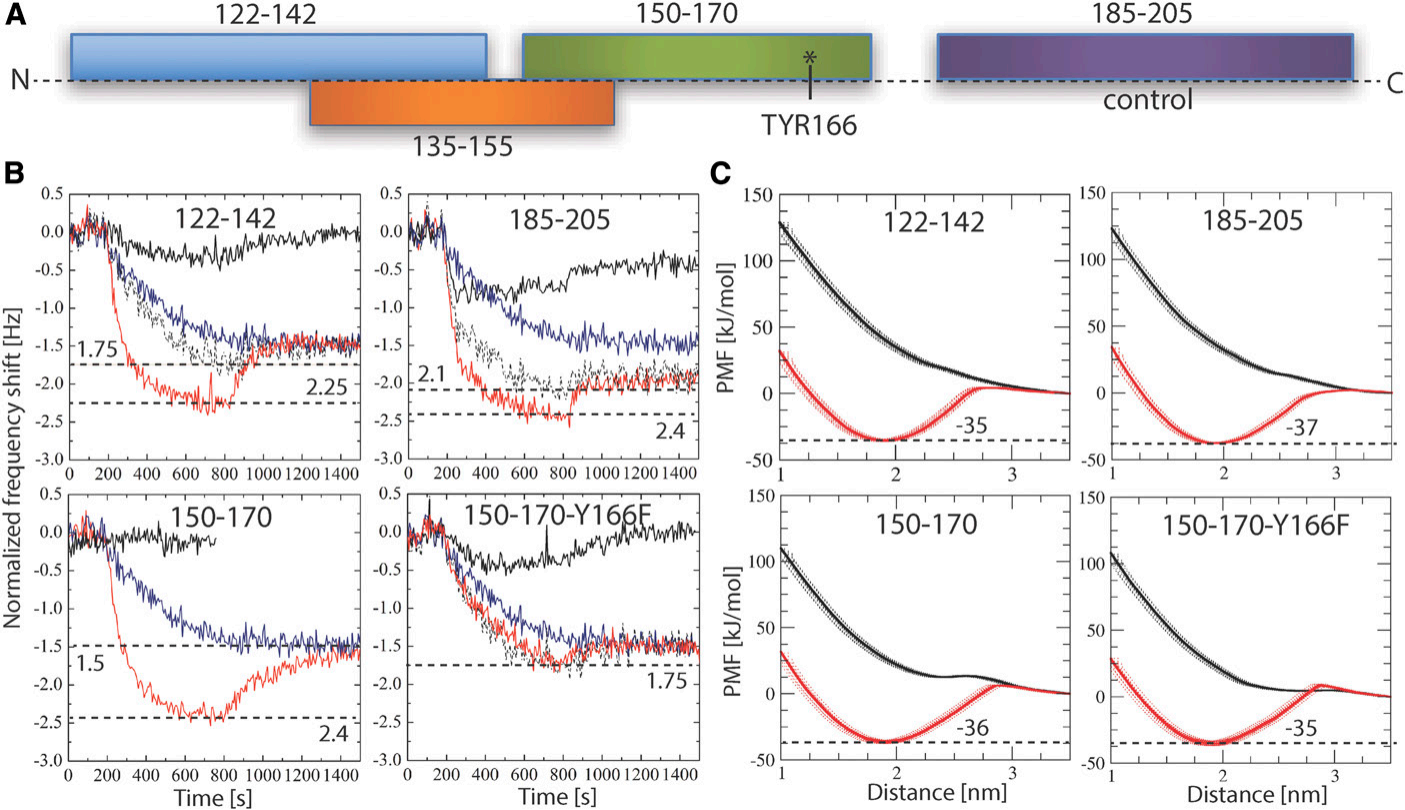
A: Schematic illustration showing the peptides studied along the sequence of apoA-I. B: QCM responses for the pure LCAT (blue curve) and peptides (black curves) on the surface of the POPC bilayer. Surface responses of LCAT in conjunction with peptides are shown in red. The sum curve of pure LCAT and peptide measurements is marked as a dashed black curve. The horizontal dashed lines mark the minimum responses for different measurements, including their value. The result for peptide 135-155 is not shown, but the profile is similar to the case of peptides 150-170 (see supplementary Fig. S3). C: PMF calculations for different peptides in coil (black) or helical (red) secondary structure as a function of distance from the center of a lipid bilayer. The binding free-energies for the peptides (kilojoules per mole) are marked next to the dashed black lines.

To gain additional mechanistic insights regarding the interactions of LCAT with lipids and the possible role of apoA-I in this, we studied the binding of LCAT to a lipid bilayer with and without apoA-I-derived peptides by employing the QCM technique. The apoA-I-derived peptides were selected from the previously proposed LCAT-activation region (26) and were based on the apoA-I amino acids of 122-142, 135-155, and 150-170 (**Fig. 4A**). In addition, a control peptide was chosen from the region of apoA-I comprising amino acids 185-205, which is not involved in the activation of LCAT. The effect of mutation Y166F on the properties of peptides 150-170 was also studied because it has been demonstrated that this mutation hampers the activity of LCAT (56).

The QCM results in Fig. 4B indicate that LCAT binds to the POPC membrane without apoA-I, agreeing with previous experimental studies and our simulation results showing that LCAT stays at the lipid membrane utilizing the tunnel-opening nonpolar amino acids or membrane-binding region in the attachment. The results also pointed out that some of the apoA-I-derived peptides did not interact with lipids, namely, only peptides 122-142, 185-205, and 150-170-Y166F were found to bind to the lipid bilayer (see Fig. 4B, solid black curve). The combined interaction studies of LCAT and peptides revealed that peptide regions 122-142, 135-155, and 150-170 clearly increased the affinity of LCAT to the lipid bilayer (Fig. 4B). Results also showed that peptides 185-205 and 150-170-Y166F did not increase the lipid bilayer affinity of LCAT.

As peptides 150-170 and 135-155 did not interact with the lipid surface individually, but still increased the overall binding of LCAT, we hypothesized that the putative apoA-I interaction site of LCAT could induce amphipathic α-helical structures for the peptides driving stronger lipid interaction due to increased lipophilicity of the LCAT-peptide complexes. Another hypothesis considered the possible role of the peptides in changing the conformation of the lid from the closed to the open state, enabling a stronger interaction of LCAT with lipids.

To test the first hypothesis, we calculated the PMF profiles for each peptide when they are transferred from the water phase to the lipid-water interface utilizing CG simulations. We carried out calculations with both coil and α-helix secondary structures to investigate the role of the secondary structure of apoA-I-derived peptides regarding the membrane interactions. The results depicted in Fig. 4C indicate that all peptides can bind to the surface of the lipid bilayer if they adopt α-helix conformation. However, none of the peptides showed a free-energy minimum within the membrane region in the coil form. Therefore, for a peptide region to induce stronger binding of LCAT to the lipid membrane surface, it must fully or partly adopt an amphipathic α-helical secondary structure to interact with the unknown apoA-I interface of LCAT.

**Fig. 5.**
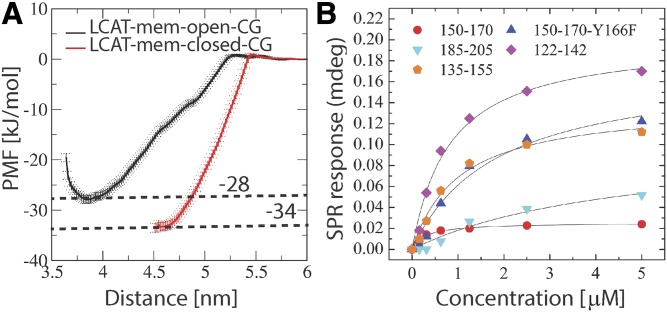
A: PMF profiles for LCAT as a function of distance from the center of the lipid bilayer. The transfer free-energies of LCAT from water to the lipid bilayer surface are marked next to the dashed lines (kilojoules per mole). B: Surface plasmon resonance peak responses for different apoA-I-derived peptides as a function of LCAT concentration.

To assess the possibility of the second hypothesis, we calculated the binding free-energies for LCAT in open and closed states. As shown by the PMF profiles in **Fig. 5A**, the lid-open state (ΔG_water→lipid_ = −28 ± 1 kJ/mol) is not able to bind so strongly to lipids when compared with the lid-closed state (ΔG_water→lipid_ = −34 ± 1 kJ/mol), although the previous findings indicated a deeper lipid interaction for the open state (Fig. 3B). The value for the closed state is well in agreement with existing experimentally determined dissociation constants giving binding a free-energy value of −36 kJ/mol for LCAT when interacting with small unilamellar vesicles in the absence of apolipoproteins (22).

To also elucidate the specificity of different peptide regions against LCAT, we carried out MP-SPR experiments to determine dissociation constants (*K_d_*) for the LCAT-peptide complexes. The peptides were attached to streptavidin-coated gold sensors via a biotin anchor and LCAT was flowed over the anchored peptides to measure the *K_d_*s using a Langmuir model (see more details in the Materials and Methods). From the MP-SPR results (Fig. 5B, **Table 2**), it can be deduced that the three strongest affinities of LCAT were with peptide regions 150-170, 122-142, and 135-155, while the lowest affinities were against peptides 185-205 (control) and 150-170-Y166F. These results suggest that LCAT possesses a more specific binding site for peptides 150-170, 122-142, and 135-155. This is also corroborated by the QCM results, which correlate well with the affinity values obtained from SPR measurements (Table 2).

**TABLE 2.** MP-SPR derived dissociation constants (*K_d_*) and *R_max_* values for different LCAT-peptide complexes

Peptide	*K_d_* (μM)	*R_max_*	QCM Difference*^a^*
122-142	1.0258	139.1	−0.5
135-155	0.8193	301.0	−1.0
150-170	0.2903	25.4	−0.9
150-170-Y177F	1.8442	174.4	0
185-205	4.6901	103.4	−0.3

a The QCM response differences between the combined LCAT+peptide and the sum of separate (LCAT and peptide alone) measurements are given.

### The acyl-intermediate of LCAT lures CHOL molecules to the active site

The entry of lipids to the active site of LCAT is still mechanistically an unknown process (14, 26). Especially, the possible direct role of apolipoproteins is unknown. To gain additional insight into this matter, we calculated PMF profiles for DOPC and UC molecules when they were pulled from the lipid matrix to the active site of LCAT. PMF calculations were carried out utilizing the equilibrated conformation of LCAT in the LCAT-mem-open-AA simulation. We assumed that the lid should be in the open state before entry of ligands.

**Fig. 6.**
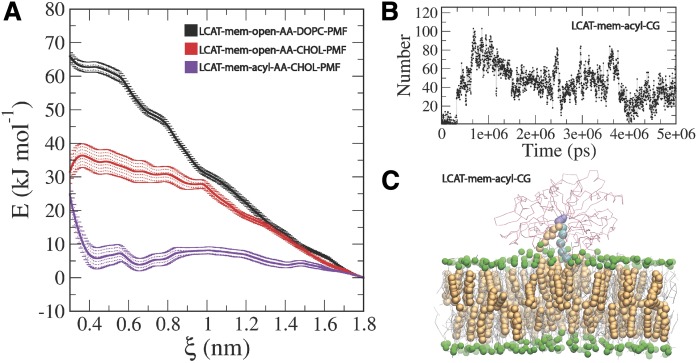
A: PMF profiles for DOPC and UC calculated in a LCAT-mem-open-AA system. In addition, the PMF profile for UC in a LCAT-mem-acyl-AA system is shown. B: The number of contacts between the oleate chain beads linked to SER181 and CHOL beads as a function of time. C: Snapshot from a LCAT-mem-acyl-CG simulation showing the location of the UC molecules in the active site of LCAT. CHOL molecules are rendered as orange spheres, DOPC molecules as gray sticks, the phosphate beads of DOPC as green spheres, LCAT backbone atoms as red sticks, the oleate chain beads linked to SER181 as cyan spheres, and SER181 as violet spheres.

The results in **Fig. 6** indicate that the free-energy costs of pulling DOPC or UC molecules from the lipid matrix to the active site are approximately 65 ± 2 kJ/mol and 35 ± 4 kJ/mol, respectively. The transfer free-energy cost of DOPC matched well with the experimental LCAT activation energies that ranged from 53 to 76 kJ/mol measured for HDL-LCAT complexes comprised of POPC or DOPC lipids (57, 58). Next, we asked whether the acylation of SER181 decreases the free-energy cost needed for UC to enter the active site. Thus, we constructed an atomistic simulation system where SER181 was acylated and the lid was in the open state (LCAT-mem-acyl-AA). Surprisingly, we found that the acyl-intermediate of LCAT did not only decrease the free-energy cost, but rendered the entry of UC to the active site almost favorable, reflected by the transfer free-energy value of 4–7 kJ/mol (Fig. 6A). To investigate the dynamics of this process further, we carried out a CG simulation with acylated SER181 (LCAT-mem-acyl-CG). The simulation trajectory revealed that after approximately 1.6 μs (scaled MARTINI time, see Table 1) a CHOL molecule spontaneously diffused to the active site (Fig. 6C, supplemental Movie S5), agreeing with our free-energy calculations. This was also registered by calculating the contacts between the side chain atoms of SER181 and the hydroxyl group of UC molecules (see Fig. 6B). In addition, a closer inspection unveiled that the exchange of CHOL molecules between the active site of LCAT and the lipid membrane can occur if the esterification reaction does not take place (supplemental Movie S6).

## DISCUSSION

In the present study, we have studied the interactions of LCAT with lipid surfaces and apoA-I-derived peptides to gain novel information regarding the molecular mechanism behind the LCAT-catalyzed CHOL esterification taking place at the surface of lipoprotein particles. To achieve this, we employed extensive atomistic and CG molecular dynamics simulations, as well as experimental surface-sensitive biophysical methods.

The simulation results showed that the lid region of LCAT forms a coil secondary structure accompanied by relatively high residual fluctuations when compared with the other structural parts of LCAT. The fact that the lid regions are partly missing from the X-ray structures of LCAT agrees with our simulation results showing a highly mobile region with no definite structural conformation (32–34). Another important finding was that the highly dynamic lid-loop shields the nonpolar amino acids located at the tunnel opening from the water. Moreover, we suggest that when the lid-loop undergoes the conformational change from the closed to the open state, two salt bridges are broken and four intra-lid hydrogen bonds are formed. These findings support the view that the lid functions as a dynamic gate regulating the access of PLs and CHOL molecules into the active site. In general, lipases are known to possess open and closed states (35, 59, 60). For example, the X-ray structures of pancreatic lipase indicate that the opening of the active site is accompanied by considerable structural changes of the lid region governed by nonpolar contacts, hydrogen bonds, and salt bridges (61). Although we have studied a structurally different enzyme, these findings are consistent with our results. Based on the current findings, it is tempting to speculate that the closed state of LCAT is stabilized in water by the lid-mediated shielding of the nonpolar amino acids at the active site tunnel opening and the presence of two salt bridges. It is possible, therefore, that the entropic cost associated with conformational switching from the closed to the open state in water mainly arises from exposing the lid-covered nonpolar amino acids to aqueous surroundings. After LCAT becomes bound to lipoprotein particles, presumably this entropic cost decreases because the nonpolar residues at the tunnel opening may become buried in a hydrophobic lipid matrix, as shown by our atomistic and CG simulations. Thus, it is possible to hypothesize that this could be one of the reasons why the lid-open state could be favored when LCAT is bound to lipoprotein particles. Yet, CG simulations revealed that LCAT can strongly interact with lipids, even in the lid-closed state, which was accompanied by a greater distance between the α/β hydrolase domain and the lipid bilayer surface. This conformation does not allow the burial of the tunnel-opening nonpolar amino acids in lipids, which we propose to be a prerequisite for the entry of lipids to the active site.

Interestingly, according to our simulations, UC molecules prefer to accumulate next to the membrane-penetrating region of LCAT. This finding may be due to packing defects between LCAT and PLs. The tendency of UC molecules to concentrate adjacent to the tunnel-opening nonpolar amino acids indicates its important role in regulating the accessibility of UC molecules into the active site over PLs. This could be one of the reasons why LCAT is the sole enzyme among lipases catalyzing the esterification of UC molecules. This issue can be further clarified by examining the lipid surface interaction of other lipases.

Our QCM measurements revealed that the peptides derived from the LCAT-interaction region of apoA-I increased the binding of LCAT to lipid surfaces, while the control peptides 185-205 and the mutant peptides 150-170-Y166F did not facilitate the LCAT interaction with lipids. These findings agree with previous experimental reports showing that apoA-I increases the binding of LCAT to lipids compared with apolipoprotein-free small unilamellar vesicles (14, 22). Earlier it has been shown that the apoA-I mutation, Y166F, decreases the surface binding and activity of LCAT, which is in agreement with our experimental results (56, 62). In general, many studies have pointed out that the charged and polar amino acids located in the central parts of apoA-I are important in LCAT activity (26, 31). One of our unanticipated findings was that, although some of the peptides did not interact with lipids alone, they still contributed strongly to the lipid attachment of LCAT. Namely, peptides 135-155 and 150-170 did not interact with lipids at all, and peptides 122-142 showed only a modest interfacial activity. Nevertheless, these peptides greatly increased the binding of LCAT to lipid surfaces when compared with the more surface-active peptides 150-170-Y166F and 185-205. Interestingly, the computational free-energy calculations revealed that all the peptides studied must adopt fully or partially amphipathic α-helical conformations before they can interact with lipid surfaces. It can thus be suggested that the LCAT-activating central region of apoA-I may adopt water-exposed loop conformations before interacting with LCAT. The interaction of loop structures with LCAT could be followed by a conformational change from the coil to α-helix leading to stronger binding of LCAT to HDL particles. The MP-SPR results support this view because it was shown that the apoA-I-derived peptides can bind to LCAT without the presence of a lipid surface. Further, based on the free-energy calculations of LCAT, it is evident that apoA-I-derived peptides do not increase the binding of LCAT-peptide complexes to lipid surfaces by merely changing the lid from closed to the open state because the lipid binding free-energy for the open state of LCAT was lower compared with the closed state. Overall, these findings strengthen the results shown in the previous experimental studies demonstrating that the central region of apoA-I possesses much lower α-helical stabilities and, thus, an increased tendency to form looped structures compared with other regions (56, 63, 64).

The free-energy calculations produced in the present study support the view that the entry of lipids into the active site of LCAT likely occurs without the direct involvement of apoA-I. One major finding supporting this view was that the calculated transfer free-energy of DOPC from the lipid bilayer into the active site (65 kJ/mol) is congruent with the experimentally determined activation energies of LCAT in reconstituted HDL particles with varying PL species (53–76 kJ/mol) (57, 58). This result is also consistent with the data demonstrating that the apolipoprotein content of HDL particles does not affect the activation energy of LCAT (65). The second major finding was that the acylation of SER181 renders the transfer free-energy of CHOL much lower when compared with the nonacylated case (4–7 kJ/mol vs. 35 kJ/mol). With respect to this finding, we observed that UC molecules spontaneously diffused into the active site in CG simulations when SER181 was acylated. The CG-simulations further revealed that the exchange of UC molecules in the active site took place in 20 μs. Thus, the atomistic free-energy calculations and CG simulations showed that the diffusion of lipids, especially in the case of UC molecules, to the active site of LCAT is not directly assisted by apoA-I. Interestingly, a recent research study revealed that molecular agents targeted for the lipid binding site of LCAT can increase the *V_max_* of LCAT (66). Surprisingly, the compound also activated LCAT deficiency causing mutants, which raises hopes for treating these disorders in the future. It was identified with molecular modeling that the LCAT-activating compound A formed a hydrophobic adduct with CYS31 located in the active site. Thus, one reason for the higher LCAT activity could be that the compound A renders the active site of LCAT energetically more favorable to PLs to diffuse in. In other words, the drug lowers the activation energy of LCAT, which is essentially covered by the transfer of a PL from the lipid monolayer to the active site.

To conclude, our results suggest that the initial binding of LCAT to the lipoprotein surface occurs with the help of nonpolar amino acids in the membrane-binding domain. The initial lipid interaction of LCAT is likely followed by an as yet unknown interaction with apoA-I, which enables the opening of the lid and the conformational change of LCAT to the configuration where the tunnel-opening nonpolar amino acids become buried in lipids. In addition, free CHOL molecules are attracted adjacent to the tunnel opening. Meanwhile, amino acids 135-170 of apoA-I increase the binding of LCAT to lipoproteins. Thus, apoA-I enables the diffusion of PLs to the active site via conformational and structural changes, which leads to the formation of the acyl-intermediate of LCAT. This, in turn, greatly facilitates the diffusion of UC molecules to the active site without the direct involvement of apoA-I. The evidence from this study suggests that the role of apoA-I and other apolipoproteins in activating LCAT is to adjust the conformation of LCAT with respect to the lipid surface, increase the binding of LCAT, and drive the lid to the open state. The reasons for varying LCAT activation potencies of different apolipoproteins could be speculated to arise from their different capacity to control these features (57). What is highly surprising is that the apoA-I-derived peptides can increase LCAT binding to lipids, even if some of them do not interact with lipids individually. These findings encourage further investigations and modifications of these peptides aiming to increase the activity of LCAT.

It should be noted that our simulated LCAT models do not include the amino acids 1-20 and 398-422 of the N- and C-terminal ends, respectively. This was because of the high uncertainties associated with these parts based on the published X-ray structures. Further, according to the hydrogen-deuterium exchange measurements carried out by Manthei et al. (33), these structural regions of LCAT are highly dynamic and solvent exposed, which renders their structure even more difficult to assess. Earlier it was shown by Vickaryous et al. (67) that the first few N-terminal amino acids are important for the activity of LCAT. In the same study, it was shown that the HDL and LDL binding was unaffected by the N-terminal deletion mutants of LCAT (Δ1 and Δ2). Recently however, during the preparation of our article, a report was published that the deletion of amino acids 1-20 and 398-422, as well as the point mutations W48A and L70S, decreased the binding of LCAT to the HDL particles with apoA-I or mimetic peptides the most (33). The highly decreased binding of W48A and L70S mutants to HDL particles is in good accordance with our simulation results showing that residues W48 and L70 interact with lipids and stay buried in the lipid acyl chain region in the lid-open state. In addition to the lipid-interacting hydrophobic amino acids reported here, it is also possible that the N- and C-terminal parts can interact with lipids, apoA-I, or both and further enhance LCAT binding to the surface of HDL particles. It is also plausible that the membrane-binding domain together with the N- and C-terminal ends interact with the molecular components of HDL in a manner that adjusts the active site tunnel opening closer to lipids, enabling lipid entry after the lid is in the open state. Nonetheless, our free-energy calculations show that the binding of LCAT to the lipid bilayer in its closed-lid state is well in agreement with the experimentally detected binding free-energy for LCAT binding to small unilamellar vesicles without the presence of apolipoproteins (−34 kJ/mol vs. −36 kJ/mol, respectively) (22). Based on this information, it is tempting to hypothesize that the N- and C-terminal ends are mainly responsible for the interaction with apoA-I and other apolipoproteins after the initial attachment of LCAT to the lipid moiety of HDL particles via the membrane-binding domain.

Another limitation of our study is that we have only studied LCAT on planar lipid surfaces (mimicking discoidal-HDL particle surfaces). As a result, we do not understand the effect and magnitude of lipoprotein particle curvature, size, and type on our results; although it has been shown that these features modulate the activity of LCAT (68).

Our study provides a novel framework for the exploration of LCAT activation by apolipoproteins, apolipoprotein mimetic peptides, and other pharmacological compounds. Eventually, the approaches presented in this study may lead to the development of more advanced apolipoprotein mimetic peptides that could be used in the treatment of different metabolic disorders, such as dyslipidemia and LCAT deficiencies (69, 70). However, the beneficence of pharmacological compounds aiming to promote the activity of LCAT in the treatment of CHD is still under debate. Therefore, further investigations are needed to clarify the role of LCAT in the development of CHD before pharmacological interventions.

## Supplementary Material

Supplemental Data
