# Supplementary material for: Interaction of lecithin:cholesterol acyltransferase with lipid surfaces and apolipoprotein A-I-derived peptides
Source: J Lipid Res. 2018 Feb 8;59(4):670–83. doi: 10.1194/jlr.M082685 (PMC5880497; doi:10.1194/jlr.M082685)
Supplement: Supplemental Data [file supp_59_4_670__index.html]

Interaction of lecithin:cholesterol acyltransferase with lipid surfaces and apolipoprotein A-I-derived peptides — Supplemental Data 

# Interaction of lecithin:cholesterol acyltransferase with lipid surfaces and apolipoprotein A-I-derived peptides

## Supplemental Data

- Supplemental Figure S1 (.pdf, 2.6 MB)
- Supplemental Figure S2 (.pdf, 1.3 MB)
- Supplemental Figure S3 (.pdf, 407 KB)
- Movie Legends (.pdf, 20 KB)
- Movie S1 (.mpg, 15.8 MB)
- Movie S2 (.mpg, 1.8 MB)
- Movie S3 (.mpg, 35.3 MB)
- Movie S4 (.mpg, 1.7 MB)
- Movie S5 (.mpg, 11.6 MB)
- Movie S6 (.mpg, 15.7 MB)
