# Supplementary material for: Interaction of lecithin:cholesterol acyltransferase with lipid surfaces and apolipoprotein A-I-derived peptides
Source: J Lipid Res. 2018 Feb 8;59(4):670–83. doi: 10.1194/jlr.M082685 (PMC5880497; doi:10.1194/jlr.M082685)
Supplement: Supplemental Data [file 10.1194_M082685_jlr.M082685-1.pdf]

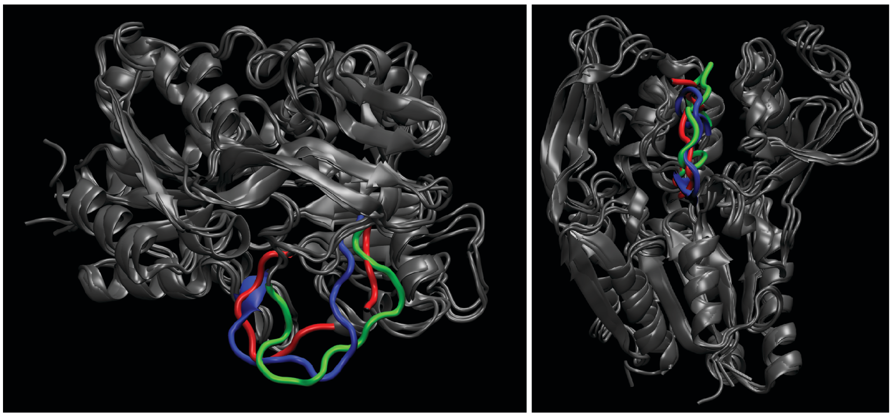

Figure S1 The superimposed structures of LCAT showing the conformation of the lid-region in the X-ray structure of 5TXF (red), before (blue) and after (green) LCAT-water-closed-AA simulation, respectively.
