# Supplementary material for: Interaction of lecithin:cholesterol acyltransferase with lipid surfaces and apolipoprotein A-I-derived peptides
Source: J Lipid Res. 2018 Feb 8;59(4):670–83. doi: 10.1194/jlr.M082685 (PMC5880497; doi:10.1194/jlr.M082685)
Supplement: Supplemental Data [file 10.1194_M082685_jlr.M082685-7.pdf]

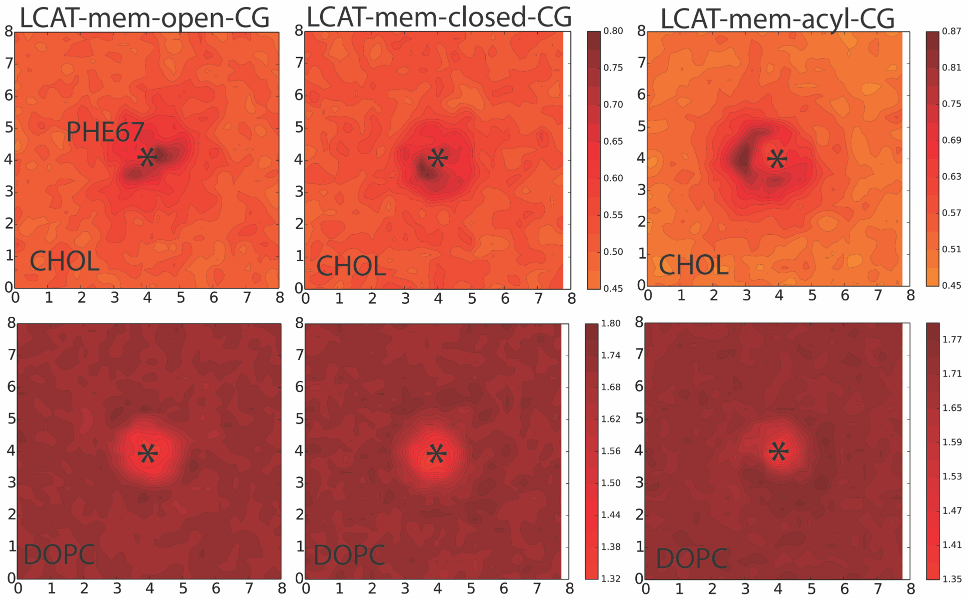

Figure S2 2D-number density maps for CHOL and DOPC molecules in the coarse-grained LCAT-mem simulations. The center of mass of PHE67 is marked by a star in the density maps showing the location of the membrane penetrating region of LCAT.
