# Supplementary material for: Interaction of lecithin:cholesterol acyltransferase with lipid surfaces and apolipoprotein A-I-derived peptides
Source: J Lipid Res. 2018 Feb 8;59(4):670–83. doi: 10.1194/jlr.M082685 (PMC5880497; doi:10.1194/jlr.M082685)
Supplement: Supplemental Data [file 10.1194_M082685_jlr.M082685-3.pdf]

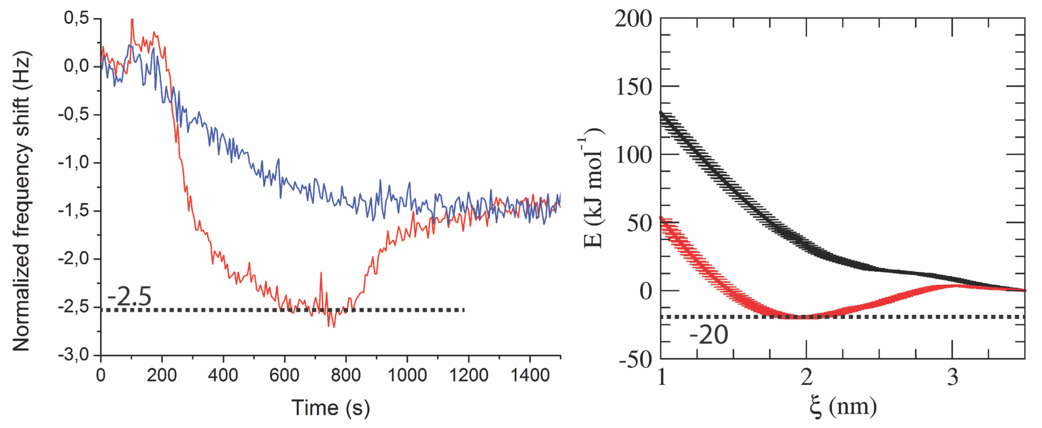

Figure S3 Left: QCM result for the peptide 135-155 (Left). Blue and red curves indicate the pure LCAT and combined LCAT+peptide measurements, respectively. The minimum response is marked with the black dashed line. Right: Potential mean force profiles for the peptide 135-155 with the coil black) and α-helical secondary structure (red).
