# Supplementary material for: Interaction of lecithin:cholesterol acyltransferase with lipid surfaces and apolipoprotein A-I-derived peptides
Source: J Lipid Res. 2018 Feb 8;59(4):670–83. doi: 10.1194/jlr.M082685 (PMC5880497; doi:10.1194/jlr.M082685)
Supplement: Supplemental Data [file 10.1194_M082685_jlr.M082685-9.pdf]

Supplementary movies

Movie S1 LCAT-mem-open-AA simulation showing the burial of non-polar amino acids (white spheres) in lipids in the lid-open state. The domains of LCAT are colored according to Figure 1. In addition, the lid region is colored with blue. The green spheres represent the phosphate groups of DOPC molecules.

Movie S2 LCAT-mem-open-CG showing the burial of non-polar amino acids (white spheres) in lipids in the lid-open state. The domains of LCAT are colored according to Figure 1. The greenish spheres represent the phosphate groups of DOPC molecules.

Movie S3 LCAT-mem-closed-AA simulation showing the burial of non-polar amino acids (white spheres) in lipids in the lid-closed state. The domains of LCAT are colored according to Figure 1. In addition, the lid region is colored with blue. The green spheres represent the phosphate groups of DOPC molecules.

Movie S4 LCAT-mem-closed-CG simulation showing the burial of non-polar amino acids (white spheres) in lipids in the lid-closed state. The domains of LCAT are colored according to Figure 1. The greenish spheres represent the phosphate groups of DOPC molecules.

Movie S5 LCAT-mem-acyl-AA simulation showing the entry of cholesterol (orange spheres) to the active site of LCAT (red sticks). The green spheres represent the phosphate groups of DOPC molecules. The oleate chain linked to SER181 is shown as cyan spheres.

Movie S6 LCAT-mem-open-AA simulation showing the exchange of cholesterol (blue and violet spheres) molecules in the active site of LCAT (red sticks). The green spheres represent the phosphate groups of DOPC molecules. The oleate chain linked to SER181 is shown as cyan spheres.
